# Developing the environmental and lifestyle exposure assessment (ELEA) tool for cancer epidemiology research in low resource settings

**DOI:** 10.7189/jogh.06.020307

**Published:** 2016-12

**Authors:** Eleonora Feletto, Joachim Schüz, Freddy Sitas

**Affiliations:** 1 Section of Environment and Radiation, International Agency for Research on Cancer (IARC), Lyon, France; 2 Cancer Research Division, Cancer Council NSW, Woolloomooloo NSW, Australia; 3 University of Sydney, School of Public Health, NSW, Australia; 4 University of NSW, School of Public Health and Community Medicine, NSW, Australia

Globally, cancer incidence has been predicted to increase by 61% from 2008 to 2030 [1] presenting new health challenges for clinicians, researchers, prevention specialists and policy makers. Moreover, the largest increase is expected in countries with a low human development index, where cancer incidence is predicted to increase by 93% [1], mainly due to demographic shifts and the changing prevalence of risk factors [2]. Existing evidence, mainly from studies in developed and highly resourced countries, indicates that the reduction of known modifiable risk factors associated with cancer, and other non–communicable diseases (NCDs), could lower incidence by approximately 30–50% and is critical to cost–effective cancer control [3]. As a result, efforts are being made to actively reduce the burden of cancer (and associated NCDs) through prevention. However, associations between risk factors and cancer outcomes in low resource settings remain largely theorised [4]. This is largely a result of the lack of high quality local information available to guide cancer control programs. The far richer genetic diversity seen in populations in low resource settings, particularly from the African continent, would render additional findings in this area both novel and insightful [5].To close this information gap we propose that action is best taken by developing a sound, systematic approach to measuring leading and emerging risk factors to guide future cancer control programs. This would generate local evidence in low resource settings complementing but not replacing targeted research as well as raising awareness and providing information from which appropriate pathways for prevention and care could be developed [2,4].

Here we summarise existing evidence and highlight factors which should be considered in the development of an easy–to–use, cost–effective and standardised data collection process to measure risk factors in low resource settings. Thus, we present an illustrative synthesis of the existing work measuring cancer risk factors in low resource settings to determine how to best facilitate future research. We identified studies covering data harmonisation, standardisation or pooling in cancer epidemiology with a focus on either risk factor measurement or low resource settings.

Our findings confirmed that the ideal method to assess risk factors, even in low resource settings, is a large prospective cohort study despite the significant investment required. Successful cohort studies exploring risk factors produce more robust results ascertaining causation with influential outputs due to their dimension and data richness [6–9], and illustrate the benefit of country–specific data for specialised intervention programs [10]. In low resource settings, India, China and Mexico have succeeded in establishing large scale cohort studies [6–8]. However, several low resource settings have restricted disease diagnostic infrastructure or death notification so the capacity to establish cohort studies is limited. Ideally, large cohort studies should be replicated to provide more definitive answers regarding the geographic heterogeneity of cancer risk factors, but these are often impractical. Rather, cancer risk factor studies in low resource settings are usually single–cancer focused retrospective case–control studies of limited duration or for a single exposure type, for example oesophageal cancer in Iran [11]. Nevertheless these studies often indicate significant heterogeneity regarding known cancer risk factors and sometimes the role of unique risk factors [12].

Photo: IARC / R. Dray

Significant work using data harmonisation and standardisation principles also exists in cancer epidemiology [13–15]. Key multinational large–scale studies illustrate the process of standardisation and harmonisation on a large scale. For instance the European Prospective Investigation into Cancer and Nutrition Study, a prospective cohort study, includes the development of a standardised dietary method of data collection which has undergone numerous modifications to facilitate standardisation, calibrate results from multiple sites and reduce the potential error [14,15]. On the other hand, the World Health Organization STEPwise approach focuses on collecting consistent data between and within countries with flexibility of use across all settings, including low resource settings, and collected using standardised tools to measure eight behavioural and biological NCD risk factors [9]. In addition, there are a number of multicentre national projects using similar principles of harmonisation and standardisation from which we saw the progression to online project management and increased flexibility in survey content to improve data collection [6–8]. However, large–scale projects are resource intensive and the data collection tools are often lengthy and context specific.

We saw evidence of standardised project management though efficient utilisation of networks, resources and investment in building local research capacity [9,14,16]. Cohort studies rely on a systematic collection of disease outcomes either via hospital admissions, disease registers or death notification. In low resource settings, it was more feasible to systematically collect information from consecutive series of cases and controls from tertiary hospitals or other relevant institutions for retrospective assessment of cancer risk factors [11,12]. However in many cases, regardless of the study design, centralised project management was used to facilitate standardisation and harmonisation by guiding the gathering and checking of data and consistently implementing changes to data collection across sites. Understandably, centralisation can also introduce a number of complex methodological, cultural, legal, ethical and custodial challenges.

Many multinational large–scale examples are complex prospective studies which require a significant research commitment from both the administering institution and participating local centres. The International Agency for Research on Cancer’s cancer registration program, the Global Initiative for Cancer Registry Development (GICR), illustrates a straightforward approach to this type of endeavour in ascertaining basic but essential population based information on cancer [17]. Since the 1960s, cancer registries have enabled the collection of cancer incidence data, also adapted to low resource settings [17,18]. These data have been used to aid political decision making and inform research into cancer control [18]. A key element to the success of the registries has been the succinct minimalist approach to collecting standardised information. Despite its challenges, the simplicity of the GICR is critical to its worldwide adoption and the availability of comparable data.

Frequently, we saw the use of data pooling, an effective coordinated approach that extended the capacity of any individual study to measure effect. The benefits were evidenced in the isolation of an association between smoking and elevated cervical cancer risk following the establishment of human papillomavirus infection as a necessary cause of cervical cancer using various case–control studies [16]. Also, occupational exposure assessment and cancer risk has used this technique as shown in the SYNERGY project which consolidated more than 350 000 exposure measurements to assess lung cancer risk and occupation globally [19]. A challenge when pooling studies is often harmonizing exposure information derived from independent studies. Theoretically if universal indicators are agreed to and utilised consistently, some challenges and study limitations could be overcome.

With this context and existing studies in mind, we can identify the need for an easy–to–use, cost–effective and systematic data collection process for cancer risk factors which could produce regionally compatible and relevant basic information in many locations. This approach is intended to complement the continuation of targeted research and capacity building in such areas and the information obtained would be useful to optimally design further targeted cancer research. We propose the Environmental and Lifestyle Exposure Assessment (ELEA) project as the first step in addressing this need. The tool development and protocol platforms for management and implementation of ELEA are outlined in **Box 1**. These incorporate a number of important issues and experiences drawn from multinational large–scale projects and other relevant studies.

Box 1 **Environmental and Lifestyle Exposure Assessment (ELEA) tool development and protocol platforms**

**Aim:** to develop an easy–to–use, cost–effective and systematic process for collecting information on the leading and emerging cancer risk factors in low resource settings (ELEA Tool) and protocols for its use (ELEA Protocols).

**ELEA tool development method**

1. Identify the key cancer risk factors together with their standardised definitions and measurements relevant to low resource settings.

2. Use a consensus approach with a group of international stakeholders experienced in cancer epidemiology and research in low resource settings to determine the factors and questions for the ELEA Tool

3. Finalise a base set of questions covering cancer risk factors and a smaller set of geographically specific questions as part of an online tool that is practical, brief and easy to administer by a trained interviewer

**ELEA protocol platforms**

Administrative platform:

• Project governance (including data access)

• Standard operating procedures

• Online management portal: electronic devices and cloud technology

Repository platform:

• Data management

• Quality control checks

• Harmonisation

• Data treatment

Implementation platform:

• Study administration

• Interviewer training

• Study design

• Study site and fieldwork management

**Feasibility testing and next steps**

• Test the feasibility of ELEA through a series of small pilot sites (case–control or case series) to check both the functionality of the ELEA Tool and ELEA Protocols.

• If successful, augment ELEA to incorporate bio–specimen sample collection and storage, and environmental measurements.

Essential to the ELEA process is ensuring the risk factors and corresponding questions chosen for inclusion are supported by the literature and by experts in the field. It is unrealistic to incorporate an exhaustive list of risk factors, but those included should be priority areas for low resource settings. Additionally, the risk factors should be potentially modifiable or important confounders, most likely to be a leading cause of cancer burden, have the greatest impact, have valid and reliable measurements tested in different populations and high response rates. In this way, the questions could both serve as a collection of retrospective information on behaviour and lifestyle as well as a potential platform for prospective data gathering. We envisage the inclusion of a base set of main questions for use across all settings covering the major cancer risk factors as well as a set of geographically specific questions (and nuanced adjuncts to the main questions, eg, tobacco chewing, specific locally brewed alcohols) to allow for local needs and differences. Universal indicators are critical in measuring these risk factors and reduce many research limitations even though their implementation is challenging. Despite this challenge, the benefits are widely acknowledged and their implementation would facilitate both data collection and comparability of country–specific findings to the international context. That is, the development of comparable cancer risk factor indicators would reduce variation in exposure measurement and permit the potential discovery of previously undetected associations [13]. ELEA strives to maintain a simple structure, making it applicable where there are limited resources. Potential ways of using ELEA would be to investigate risk factors in exploratory cancer case series studies or hospital based case–control studies. If successful, a biobank component could then be added as a special module depending on local resources.

With the advent of m–Health (mobile health: public health activities supported by mobile devices), many tools have also transitioned from paper based data collection to online platforms to allow for quicker fieldwork and real time feedback. Studies also incorporate the development of online portals as platforms for sharing measurements, frameworks, standards, policies and data sets with positive results. Although internet coverage can be unpredictable, coverage is being proactively enhanced which would aid online project administration. Online methods facilitate real time administration, flexibility and quality control which is integral to ensure consistency and data harmonisation [15]. Additionally, treating data centrally ensures consistent modifications are made. Endeavours of this nature require sophisticated data sharing and governance frameworks as well as common protocols to guide fieldwork.

Moreover, understanding the interplay of lifestyle, environmental and genetic factors is arguably critical to determining the pattern of risk factors to improve cancer control and to prioritising evidence based initiatives especially. Innovative and novel research approaches to overcome the barriers of large scale studies and build on techniques from previous successes are encouraged to advance cancer epidemiology. The first step in research innovation in cancer epidemiology must be the collection of basic but essential descriptive information on risk factor distribution to better guide more complex and complementary techniques. ELEA proposes to develop a strong platform to increase the research capacity of low resource settings and clearly address a gap in knowledge. ELEA could be used as a precursor to large–scale studies, by creating a scientific knowledge base allowing preliminary assessment of the relevance of major cancer risk factors in various settings and populations. By collating a small amount of highly relevant data per subject in a systematic manner across large samples and diverse populations, ELEA would lead to the creation of a Big Data repository, a key resource for cancer epidemiology.

With better epidemiological evidence, local leadership could have an increasing voice in local and international cancer control forums that is supported by evidence. It will function as a catalyst for international collaboration and cooperation with a focus on cancer as a development issue in low resource settings and apply knowledge to complexities of the real world. The extension to existing knowledge of cancer causes from future findings could transform approaches to cancer prevention and treatment.
